# Reconstructing the brain: from image stacks to neuron synthesis

**DOI:** 10.1007/s40708-016-0041-7

**Published:** 2016-02-24

**Authors:** Julian C. Shillcock, Michael Hawrylycz, Sean Hill, Hanchuan Peng

**Affiliations:** 1 Blue Brain Project, EPFL, 1211 Geneva, Switzerland; 2 Allen Institute for Brain Science, Seattle, WA 98109 USA

**Keywords:** Neuron, Automated reconstruction, BigNeuron, Morphometric analysis, Synthesis

## Abstract

Large-scale brain initiatives such as the US BRAIN initiative and the European Human Brain Project aim to marshall a vast amount of data and tools for the purpose of furthering our understanding of brains. Fundamental to this goal is that neuronal morphologies must be seamlessly reconstructed and aggregated on scales up to the whole rodent brain. The experimental labor needed to manually produce this number of digital morphologies is prohibitively large. The BigNeuron initiative is assembling community-generated, open-source, automated reconstruction algorithms into an open platform, and is beginning to generate an increasing flow of high-quality reconstructed neurons. We propose a novel extension of this workflow to use this data stream to generate an unlimited number of statistically equivalent, yet distinct, digital morphologies. This will bring automated processing of reconstructed cells into digital neurons to the wider neuroscience community, and enable a range of morphologically accurate computational models.

## Introduction

Neuron morphologies are fundamental to brain function, but are difficult to quantify. Many schemes have been created that capture some features of selected cell types, but they are hard to generalize to many cell types. One bottleneck is that describing the shape of a neuron requires quantitatively specifying many morphological features, examples of which are the length and branching patterns of neurites, and their spatial distribution. Another is that obtaining accurate 3D digital representations of neurons has traditionally been a slow, expensive, manual process. The public database Neuromorpho.org currently contains about 34,000 reconstructed neurons at different levels of completeness. A small fraction of such documented neurons are from mammalian nervous systems as the result of many years work by many groups. The Blue Brain project, which recently published a first draft reconstruction and simulation of a portion of rat somatosensory cortex [1], has collected around 2000 biological reconstructions using standardized protocols. However, these datasets are insufficient to map even a small part of the rodent brain, which contains of the order of 100 million neurons.

In addition, the quality of neuron morphologies produced by existing efforts, such as those stored in NeuroMorpho.org, varies widely, reflecting the different protocols used in the experiments and reconstruction. If the ambitious goals of large-scale projects like the European Human Brain project and the US BRAIN initiative are to be realized, an automated workflow is required to produce large numbers of reconstructed, biological neurons, quantitatively analyze their shapes, and generate from them the vast number of cells needed for whole brain modeling. Each stage should not only preferably be independently executable, so that many community use cases are satisfied, but also be seamlessly connected to fulfill the integrated need of larger brain modeling projects.

**Fig. 1:**
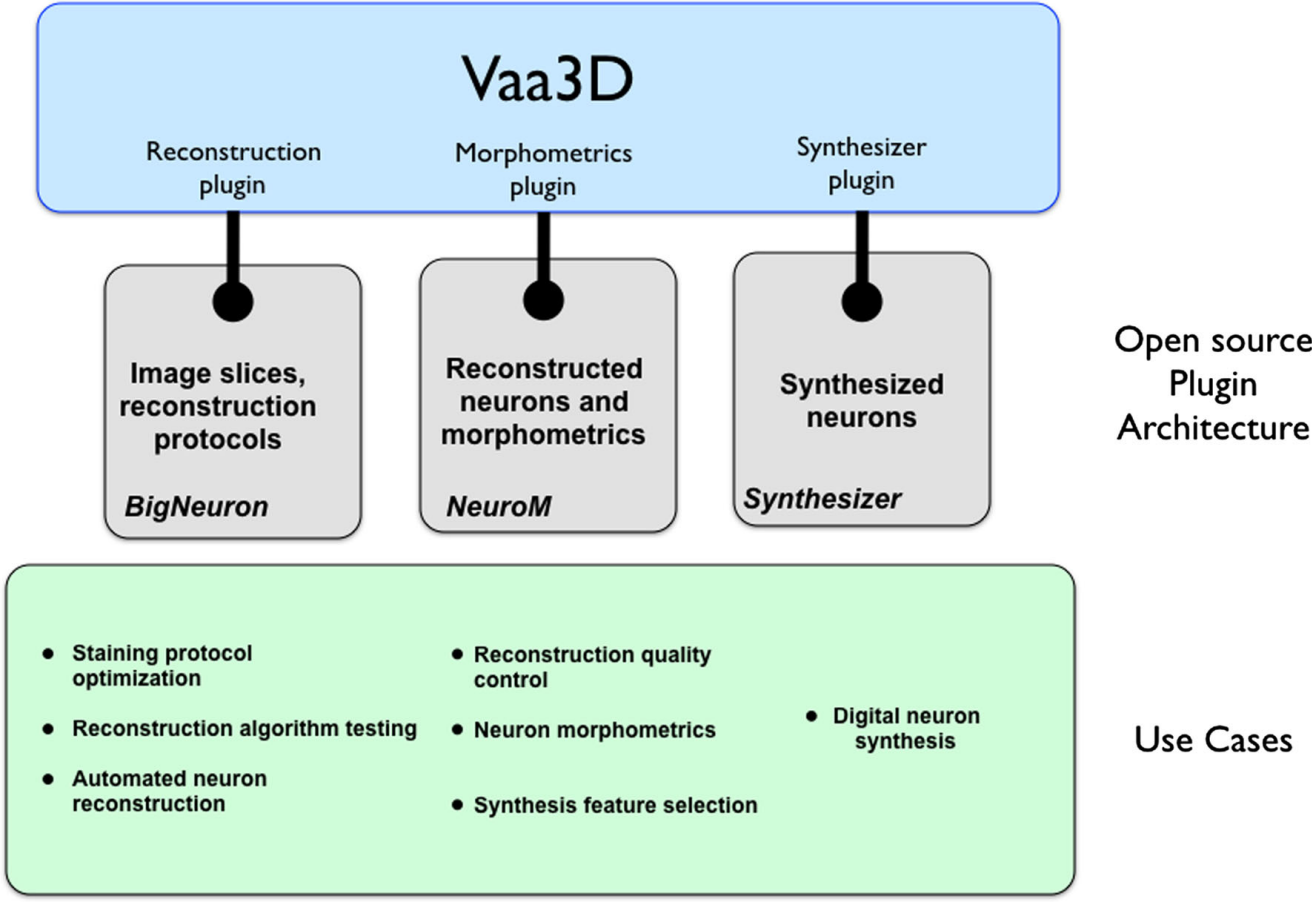
Vaa3D is a cross-platform framework that provides a plugin architecture to expose community-provided features for neuronal reconstruction, morphometric analysis, and digital neuron synthesis. Individual plugins allow users to solve many current neuron reconstruction use cases separately, or take advantage of the unified workflow that uses automated reconstruction and analysis algorithms to generate multiple digital neurons for network modeling and simulation

We believe an automated workflow such as that demonstrated in Fig. 1 would eliminate the barrier that experimentally reconstructing neuronal morphologies is labor-intensive, slow and does not scale to the generation of sufficient cells for whole brain exploration. By using open data formats and combining community-generated algorithms on a common hardware platform, such a workflow will reduce the need for expert knowledge in processing the brain slices, tracing out the neuronal shapes, and extracting the morphometric features needed for creating artificial neurons. This workflow also builds on a recent trend by large international organizations toward developing open-source software tools for the neuroscience community that can be leveraged to embed currently manual tasks into a single, seamless workflow.

There are essentially three stages in our workflow (Fig. 1):

- Reconstruction of digital 3D neuronal structures from brain slice images;
- Morphometric analysis of the digital neurons to extract quantitative features that characterize them; and
- Generation of arbitrary numbers of cells using these statistical features.

## Automated neuronal reconstruction

The BigNeuron initiative (http://bigneuron.org) [2, 3] is led by the Allen Institute for Brain Science and co-sponsored by 15 organizations across the world. The project aims to combine multiple, community-authored automated neuronal reconstruction algorithms in one open-source platform. By applying many independent algorithms to a standardized image dataset, BigNeuron will produce a potentially better estimate of the neuronal shape than any single algorithm. It also enables anyone who wants to contribute a new reconstruction algorithm to compare it with existing ones, and to test it on the large set of image slices provided.

The technical platform of BigNeuron is built upon the Vaa3D software (http://vaa3d.org) [4, 5], an open-source visualization and analysis software suite created and maintained by Janelia Research Campus of Howard Hughes Medical Institute and the Allen Institute for Brain Science. Figure 1 illustrates how the plugin architecture of Vaa3D exposes community-provided tools needed for neuron reconstruction and subsequent stages of analysis. Plugins typically are also open-source software tools that can be used individually, or combined into automated workflows. Plugins can be used to solve typical use cases in reconstruction, such as testing reconstruction algorithms on image slices, optimizing staining protocols in the laboratory, and analyzing reconstructed neurons to extract quantitative morphometric features. Eventually, Vaa3D will unify the tools needed to automate the process of using brain slice images to ultimately generate large numbers of synthesized neurons automatically.

Reconstruction algorithms are developed independently by the individual groups, who can choose to release their algorithms as plugins to the software that are then available to all. The input dataset of mouse brain images were contributed by the Allen Institute for Brain Science and other research organizations using a standardized protocol. This makes it possible to explore the effects of changes to the algorithms on the reconstruction quality, and give feedback both to the experimental groups doing the image stains and the software developers producing the image analysis plugins.

The BigNeuron initiative has run a series of hackathons in 2015 (http://alleninstitute.org/bigneuron/hackathons-workshops/) at which developers work with image reconstruction methods to make their algorithms available on the Vaa3D platform. Because the platform is open source, any member of the community can create their own plugin for their private use or take advantage of those deposited by the community. Currently, BigNeuron incorporates around 30 reconstruction algorithms that can be applied to a set of 30,000+ multi-dimensional image stacks. This has so far resulted in more than one million reconstructed neurons from different species. For mouse and other mammal brains, there are hundreds of increasingly high-quality reconstructions. The first official data release is planned for 2016 in the common swc format (http://research.mssm.edu/cnic/swc.html).

## Automated neuronal morphology analysis

Given a large number of reconstructed neurons, the next stage is to transform their 3D structures into a set of morphometric features that can be used to create digital cells. Vaa3D comes with a set of morphometric features that are consistent with the popular neuron morphometric analysis tool L-measure [6]. Vaa3D also provides neuron search and comparison tools such as BlastNeuron [7].

The Blue Brain Project (BBP) in Switzerland has recently released an open-source software tool called NeuroM (https://github.com/BlueBrain/NeuroM) that allows a user to import digitally reconstructed neurons in swc format, apply simple checks on the quality of the reconstruction, quantify a variety of features, find correlations between features, and explore the spatial distribution of the neurites. The tool is written in python and depends only on common open-source python packages. It is designed to help neuroscientists quantitatively and objectively measure neuronal features, assign neurons to stable classes, and share their results between laboratories. An initial suite of analysis functions is provided by NeuroM that allows a user to extract simple morphometric features. The neuroscience community has not yet converged on the optimal set of features needed to reliably describe neurons of any cell class [8]. Consequently, a tool that allows the measurement of a large number of morphological features is desirable. Python users can easily extend the initial functionality of NeuroM to contribute more advanced morphological measurements to the community.

Classifying neurons into distinct classes based on their morphological features has occupied many scientists since Cajal. Neurons have a large diversity of shapes, electrical behavior, and gene expression signatures that together determine the role of each neuron in the brain. Increasing quantities of genetic, physiological, and morphological data about neurons are being produced around the world. In order to make sense of this data, and to be able to communicate it intelligibly between different groups, it is necessary to assign it to widely accepted, stable cell classes. The difficulty of finding these classes has recently been illustrated by DeFelipe et al. [9] who attempted to produce an expert-independent set of categories for GABAergic aspiny or sparsely spiny cortical interneurons.

A group of 42 experts were asked to label each cell in a large set with a variety of features, and the agreement and disagreement between experts were measured. These features included geometric properties such as *intralaminar* (axonal arbor remains in the same cortical layer as the soma) or *translaminar* (axonal arbor is distributed mainly above or below the cortical layer containing the soma); *intracolumnar* or *transcolumnar*, which applies the same criterion as the previous feature but to the arbitrarily selected region delineated by a circular column of diameter 300 microns centered on the soma. The experts were also asked to assign the cells to widely recognized neuronal classes such as Martinotti, Chandelier, Neurogliaform cell, etc. Their study concluded that a purely morphological approach to neuron classification is currently not feasible, and that different investigators use mutually inconsistent schemes for classifying neurons. They also found that several experts assigned a different type to a neuron in their study than the one the experts had chosen in earlier publications involving the same neuron.

Objective classification of neurons requires standardized brain slice imaging and neuron reconstruction protocols, and a common set of morphometric features for the consistent quantifying of cell morphologies. The increasing number of morphologies being generated by BigNeuron fulfills the first requirement, and the inclusion of NeuroM in the BigNeuron extension pipeline brings a common set of quantitative analysis tools to the community. The Blue Brain project is continuing to develop NeuroM and release its improvements via the public github repository.

The final stage of the proposed unified workflow is to grow, or synthesize, digital neurons that are statistically indistinguishable from the original biological reconstructions from which the features were extracted. Currently, statistical distributions for only a few features (the total number of sections, section lengths, and bifurcation angles) are required to synthesize neurons whose *basal* *dendrites* resemble biological cells. But, the wide variation in appearance of different neuron types, and especially the variety of axonal arbor shapes, suggests that each class of cell will require its own specific set of features to reproduce it. Whereas the software for the first two stages in this workflow has been released to the community, the final stage of synthesis is still being developed at the BBP.

## Neuron synthesis

For many years, groups have been trying to find ways of accurately recreating the highly ramified shapes of neurons on a computer. For example, the Trees toolbox [10] creates dendrites by first distributing points in 3D space according to the density obtained by overlapping many examples of a given cell type, and then uses a minimal spanning tree to connect the points in a stochastic way that yet reproduces the connectivity and appearance of various neuron classes. The NetMorph algorithm [11] grows each neurite as a quasi-random walk in space where the next point is chosen by summing up forces that reflect biophysical properties such as microtubule-based neurite stiffness and biochemical processes that lead to bifurcations. Luczak [12] has used a Diffusion Limited Aggregation scheme with a spatially imhomogeneous distribution of diffusing particles within a prescribed volume to create distinct neuronal dendritic shapes.

These algorithms derive morphological features from a set of biological neurons and recreate each synthesized cell independently with little or no information about the surrounding space. Essentially, the shape of the cell, which is clearly influenced by the composition of the tissue within which it grows and the presence of other cells, is abstracted into a set of statistical distributions. This means that some biological influences on cell growth are ignored while other aspects that would arise naturally as a result of simultaneous development of many cells must be inserted manually. Neurite tortuosity, for example, likely reflects the need for neurites to wind around rather than pass through each other, and so should not need to be explicitly parametrized.

However, biological neurons do not grow in a vacuum. The cells produced by the above schemes, although they can accurately capture some neuronal shapes, inevitably intersect unrealistically when many are placed together in a limited volume of space to build a network. This has direct consequences for neuron simulations; in that the mass distribution is unrealistic and reduces the accuracy of metabolic models and distributed electric field effects. A more realistic approach is to compose a tissue by simultaneously growing many neurons within the desired volume [13–15]. The BBP synthesizer, which forms stage 3 of the workflow in Fig. 1, uses a neuron class-dependent set of statistical features obtained from the NeuroM tool to simultaneously grow a large number of cells within a user-defined volume of space. The software incorporates external boundaries, such as the pia and white matter, and internal (transparent) boundaries such as the six layers in the cortex. It is designed to capture other internal occluding structures such as the vasculature. An immediate question arises with such an approach: given that cortical gray matter is on average 70 % filled with axons and dendrites [16]: is it possible to grow a large number of neurons simultaneously? What happens if they run out of space? A necessary condition for simultaneous growth of many neurons to be feasible is that the computational cost of adding mass to the neurites should be linear in the total mass over a wide range of volume fractions, otherwise the algorithm will not scale to the size of a brain, not even that of a mouse.

We can estimate this computational cost by considering the number of calculations a program must make to grow the neurites by adding small units of mass. A naive algorithm would simply add a mass unit and check that it does not overlap with any existing mass. But this results in an O(N^2^) cost which grows prohibitively with the linear dimension of the system. A more accurate calculation can be compared to the integration of Newton’s laws for fluid elements in hydrodynamic flow. In both cases, the only forces that act on a small material element are local and must propagate across the surface of the element. In the fluid simulation, the forces between all adjacent fluid elements must be calculated and summed to provide the net force that is then integrated to find the new positions and momenta of the elements. In the synthesis case, we consider the computational cost of each growing tip having to find a space around itself to place a new piece of mass. This calculation leads to the conclusion that synthesizing a large number of neurons simultaneously is linear in the total mass of the neurons. This is a prerequisite if synthesis is to be scalable. The BBP synthesizer is planned to be integrated into the Vaa3D plugin architecture, and released to the community, thereby completing the workflow described at the beginning of this article.

The workflow of Fig. 1 emphasizes that algorithms that are developed as open-source software tools and adhere to common data standards satisfy many existing use cases relating to automatic neuronal reconstruction and morphological analysis. The common platform encourages users to collaborate toward improving tools according to community standards. And when combined in a common platform, users can leverage high-throughput data generation and feature extraction to synthesize large numbers of neurons of specified types within prescribed volumes of tissue for use in modeling and simulation. This will bring morphologically accurate neuron simulations to the wider community, and help accelerate our pursuit of understanding the mammalian brain.
